# DJ-1 deficiency attenuates expansion of liver progenitor cells through modulating the inflammatory and fibrogenic niches

**DOI:** 10.1038/cddis.2016.161

**Published:** 2016-06-09

**Authors:** L Chen, M Luo, X Sun, J Qin, C Yu, Y Wen, Q Zhang, J Gu, Q Xia, X Kong

**Affiliations:** 1 Department of Liver Surgery, Renji Hospital, School of Medicine, Shanghai Jiao Tong University, Shanghai, China; 2 School of Biomedical Engineering, Shanghai Jiao Tong University, Shanghai, China; 3 Department of General Surgery, Ninth People's Hospital, School of Medicine, Shanghai Jiao Tong University, Shanghai, China; 4 Department of Liver Diseases, ShuGuang Hospital Affiliated to Shanghai University of Chinese Traditional Medicine, Shanghai, China; 5 Department of GI Surgery, Renji Hospital, School of Medicine, Shanghai Jiao Tong University Shanghai, Shanghai, China

## Abstract

Our previous study suggested that DJ-1 has a critical role in initiating an inflammatory response, but its role in the liver progenitor cell (LPC) expansion, a process highly dependent on the inflammatory niche, remains elusive. The objective of this study is to determine the role of DJ-1 in LPC expansion. The correlation of DJ-1 expression with LPC markers was examined in the liver of patients with hepatitis B or hepatitis C virus (HBV and HCV, respectively) infection, primary biliary cirrhosis (PBC), primary sclerosing cholangitis (PSC), nonalcoholic fatty liver disease (NAFLD), cirrhosis or hepatocellular carcinoma (HCC), respectively. The role of DJ-1 in LPC expansion and the formation of LPC-associated fibrosis and inflammation was examined in a 3,5-diethoxycarbonyl-1,4-dihydrocollidine (DDC) diet-induced liver injury murine model. We also determined the ability of hepatic stellate cells (HSCs) in recruiting macrophages in DJ-1 knockout (KO) mice. The expression levels of DJ-1 were upregulated in the liver of HBV, HCV, PBC and PSC patients and DDC-fed mice. Additionally, DJ-1 expression was positively correlated with LPC proliferation in patients with liver injury and mice with DDC exposure. DJ-1 has no direct effect on LPC proliferation. Reduced activation of HSCs and collagen deposition were observed in DJ-1 KO mice. Furthermore, infiltrated CD11b^+^Gr-1^low^ macrophages and pro-inflammatory factors (IL-6, TNF-*α*) were attenuated in DJ-1 KO mice. Mechanistically, we found that HSCs isolated from DJ-1 KO mice had decreased secretion of macrophage-mobilizing chemokines, such as CCL2 and CX3CL1, resulting in impaired macrophage infiltration. DJ-1 positively correlates with LPC expansion during liver injury. DJ-1 deficiency negatively regulates LPC proliferation by impairing the formation of LPC-associated fibrosis and inflammatory niches.

The liver has a spectacular regenerative ability in response to various injuries, also known as compensatory hyperplasia, in which both existing hepatocytes and facultative liver progenitor cells are involved.^1, 2^ When the liver suffered from minor injury, the remaining hepatocytes could replicate to compensate the metabolic needs. However, during a severe or chronic insult, hepatocyte replication is insufficient to restore the hepatic function, as such, a facultative compartment of liver stem/progenitor cells (LPCs) is activated and induced to compensate for hepatic cell mass, structure, and function.^3, 4^ LPCs, generally called oval cells in rodents, carry bi-potency to differentiate into hepatocytes and biliary epithelial cells.^5^ In rodent models, LPC response (ductular reaction (DR)) can be commonly be induced either by a 2-acetylaminoflurene injection accompanied with a 2/3 partial hepatectomy or by feeding animals with a choline-deficient ethionine-supplemented (CDE) or a 3,5-diethoxycarbonyl-1,4-dihydrocollidine (DDC) diet.^6, 7, 8^

LPCs are rarely observed in the liver of healthy individuals but are ordinarily detectable in patients with nonalcoholic fatty liver disease (NAFLD), virus hepatitis and cirrhosis.^7, 9, 10^ The livers of those patients carry remarkable continuous fibrosis and inflammatory responses. In the rodent models, accumulating evidence has suggested that fibrogenic and inflammatory responses correlate tightly with LPC proliferation.^11^ Increased *α*-smooth muscle actin positive (*α*-SMA^+^) matrix-producing cells and matrix deposition always serve as a positive indicator of the augment of oval cells during liver injury.^10^ In addition, several crucial immune cells and their associated cytokines, such as lymphocytes, macrophages, interleukin 6 (IL-6), IL-22, tumor necrosis factor-*α* (TNF-*α*), TNF-related weak inducer of apoptosis (TWEAK), interferon *γ* (IFN-*γ*) and lymphotoxin *β*,^6, 12, 13, 14, 15, 16, 17, 18, 19^ have been well documented to have a vital role in promoting LPC expansion.

*DJ-1*(*PARK7*) is originally identified as a gene associated with autosomal early-onset Parkinson's disease.^20^ In the past two decades, some novel functions of DJ-1 had been elucidated linking this gene to cancer, antioxidative stress and metabolism.^21, 22, 23^ Furthermore, emerging evidence of DJ-1 in inflammation has also been reported. Macrophages with DJ-1 deficiency showed downregulation of NF-κB-targeted pro-inflammatory cytokines *in vitro*.^24^ Additionally, our recent study has demonstrated that DJ-1 is a pivotal modulator in triggering inflammatory response by targeting NADPH oxidase-mediated reactive oxygen species (ROS) generation and subsequently enhancing the secretion of pro-inflammatory cytokines, such as IL-6 and TNF-*α*, which are well known to promote LPC proliferation.^6, 25^ However, whether DJ-1 could stimulate LPC proliferation by modulating the inflammatory and fibrosis microenvironment remains elusive.

In the present study, we demonstrated that, in both patients with hepatitis B virus (HBV) infection and a DDC diet-induced murine liver injury model, the hepatic DJ-1 expression was upregulated and positively correlated with the activation of LPCs. We showed that DJ-1 is required for LPC activation because DJ-1 deficiency severely impaired the LPC expansion in DDC-induced liver injury. Although there is no direct effect of DJ-1 on LPC proliferation, we demonstrated that, by maintaining fibrogenic and inflammatory microenvironment, DJ-1 indirectly enhanced LPC expansion in a DDC-induced liver injury murine model.

## Results

### DJ-1 expression is upregulated in the livers of patients with chronic liver injury and positively correlates with the activation of LPCs

**Figure 1:**
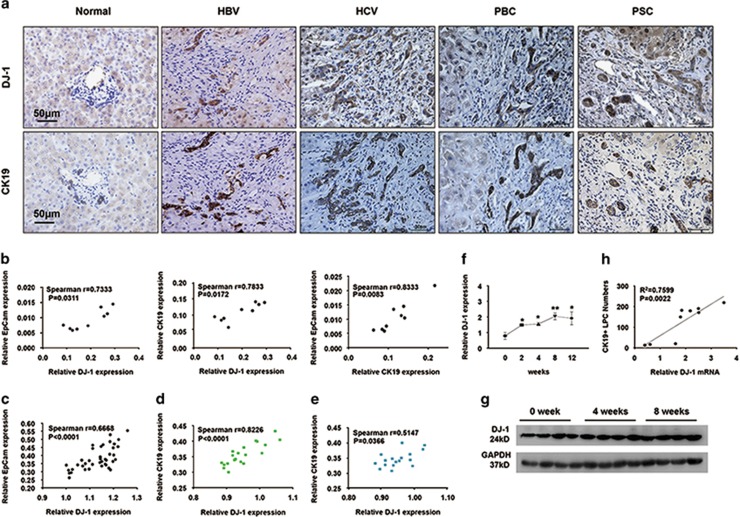
DJ-1 expression is upregulated in patients with chronic liver disease and in mice after DDC-induced LPC activation and proliferation. (**a**) Representative IHC with anti-CK19 and anti-DJ-1 antibodies of serial liver sections from normal and patients with chronic liver injury. The original magnification is × 400. (**b**) Correlation analysis of DJ-1 and CK19 in the liver tissues from HBV patients. (**c**–**e**) GEO data analysis between DJ-1 and LPC proliferation in patients with NAFLD, cirrhosis and HCC (GSE49541 and GSE17548), respectively. (**f**) The relative DJ-1 mRNA expression level was detected in WT mice after DDC diet feeding for different time periods. (**g**) DJ-1 protein level in WT mice after DDC diet feeding for 0, 4 and 8 weeks. GAPDH (glyceraldehyde 3-phosphate dehydrogenase) expression was used as a loading control. (**h**) Linear regression analysis of the DDC liver for 0, 4, and 8 weeks showed that DJ-1 mRNA levels correlated with the average number of CK19+ LPCs present in the same liver. *P<0.05 and **P<0.01. Data are presented as means±S.E.M.

Emerging evidence demonstrated that the expansion of LPCs is frequently located in the inflammatory regions of the liver in patients with virus cirrhosis.^10^ Given the pivotal role of DJ-1 in modulating inflammation, we started to examine whether the expression of DJ-1 correlates with LPC expansion. We performed immunohistochemical (IHC) analyses on the liver tissue sections of patients with chronic HBV or hepatitis C viral (HCV) infection, primary biliary cirrhosis (PBC) and primary sclerosing cholangitis (PSC) by staining CK19 (cytokeratin19, a specific LPC marker) and DJ-1. As shown in Figure 1a, the expression of both CK19 and DJ-1 was highly induced in the liver tissues of these patients compared with the normal liver control. To further confirm this correlation, the hepatic mRNA levels of DJ-1, CK19 and EpCam (epithelial cell adhesion molecule, another specific marker of LPCs) were measured by qPCR. As shown in Figure 1b, the expression of DJ-1 was highly correlated with CK19 or EpCam in the liver of chronic HBV patients. GEO data analysis suggested the positive correlation between DJ-1 and LPC proliferation in patients with NAFLD, cirrhosis and hepatocellular carcinoma (HCC) (GSE49541 and GSE17548) (Figures 1c–e).

We next sought to verify this correlation in a murine liver injury model induced by DDC diet feeding. We measured the expression changes of hepatic DJ-1 in wild-type (WT) mice fed with DDC diet. As shown in Figures 1f and g, compared with the WT mice feeding with a normal chow, both the hepatic mRNA and protein levels of DJ-1 were significantly upregulated in the mice after feeding with DDC diet for 4 and 8 weeks. Moreover, a strong correlation between DJ-1 mRNA levels and the numbers of LPCs was assessed in the livers of WT mice after 0, 4 and 8 weeks of DDC diet feeding (Figure 1h). Together, in both patients with chronic liver injury and a DDC diet-induced liver injury murine model, we demonstrate that the expression of hepatic DJ-1 was increased and positively correlated with the expansion of LPCs.

### LPC proliferation was severely impaired in DJ-1 KO mice fed with DDC diet

**Figure 2:**
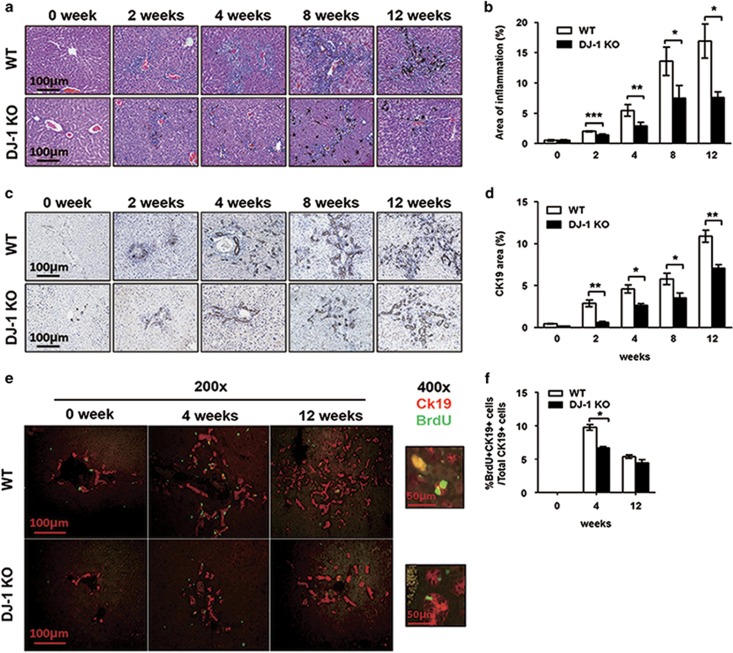
LPC proliferation in DJ-1 KO mice was decreased after DDC diet feeding. (**a**) Representative hematoxylin–eosin staining of liver tissues from WT and DJ-1 KO mice at different time periods. The original magnification is × 200. (**b**) The areas of inflammation from panel (**a**) were quantified. (**c**) Representative IHC analyses with an anti-CK19 antibody of liver tissues from mice fed with DDC diet for different time periods. (**d**) The areas of CK19+ staining from panel (**c**) were quantified. (**e**) CK19 (red)/BrdU (green) double staining of liver tissues from mice fed with DDC diet for 4 or 12 weeks with BrdU injection 2 h before killing (The original magnification of the left panel is × 200 and of the right panel is × 400). (**f**) The number of BrdU+ CK19+ double staining cells and CK19+ cells were quantified. *P<0.05, **P<0.01 and ***P<0.005. Data are presented as means±S.E.M.

As we demonstrated that there was a positive correlation between DJ-1 expression and LPC activation in the injured livers of patients and mice, respectively, the next question is whether DJ-1 contributes to LPC activation. To answer this question, we fed WT and DJ-1 KO mice with DDC diet to induce liver injury. After a short term (2 and 4 weeks) and a long term (8 and 12 weeks) of DCC diet feeding, liver sections were prepared from those mice for IHC and immunofluorescence staining. Hematoxylin–eosin (HE) staining showed increased hepatic inflammation and LPC expansion predominantly in the periportal areas after DDC exposure in a time-dependent manner in both murine genotypes (Figures 2a and b). Compared with WT mice, significant less inflammation was observed in the liver of DJ-1 KO mice (Figures 2a and b). In line with the previous findings that inflammatory response has a positive role in LPC expansion, fewer LPCs were detected in the liver of DJ-1 KO mice (Figures 2a and b). CK19 staining confirmed the reduced LPC expansion in DJ-1 KO mice compared with WT ones after DDC diet feeding (Figures 2c and d). In agreement with the less LPCs in DJ-1 KO mice, immunofluorescent double staining with bromodeoxyuridine (BrdU) and anti-CK19 showed the significantly less BrdU incorporation in the LPCs of DJ-1 KO mice compared with WT mice 4 weeks after DDC exposure (Figures 2e and f), which is also observed in a CDE model (Supplementary Figure S1a). Compared with 4 weeks of DDC exposure, the percentage of BrdU^+^CK19^+^ cells was decreased and comparable in both WT and DJ-1 KO mice after 12 weeks of DDC feeding (Figures 2e and f).

### DJ-1 has no direct effect on LPC proliferation

**Figure 3:**
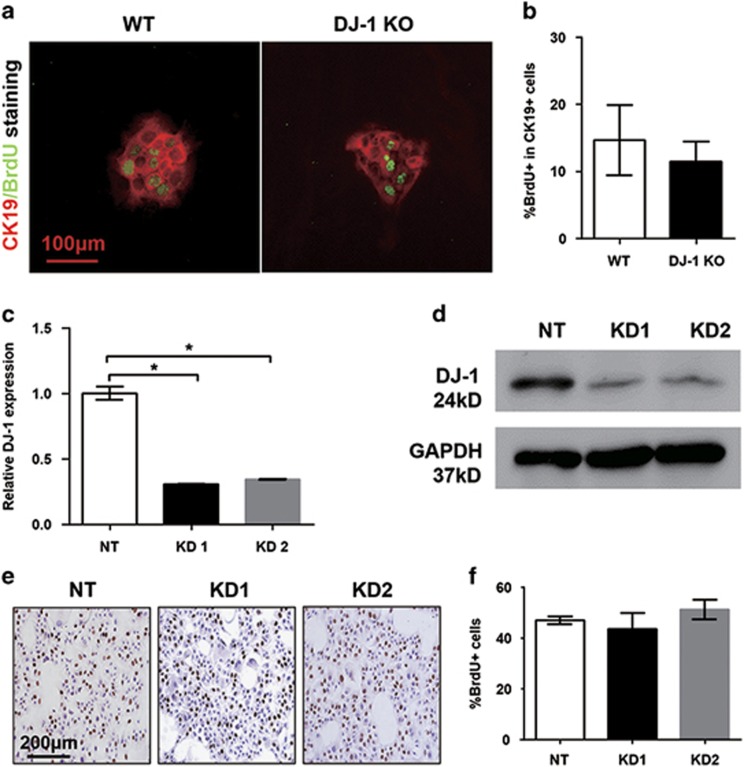
DJ-1 disruption has no effect on LPC proliferation *in vitro*. (**a**) Immunofluorescence of CK19/BrdU in LPCs isolated from WT and DJ-1 KO mice after DDC feeding 4 weeks (× 200). (**b**) The proportions of BrdU+ cells in total CK19+ cells were quantified. (**c** and **d**) Relative DJ-1 mRNA and protein level were analyzed in non-targeted cells (NT) and two established DJ-1 KD cell lines (KD1 and KD2). GAPDH (glyceraldehyde 3-phosphate dehydrogenase) was used as a loading control. (**e**) Representative IHC of BrdU staining in NT, KD1 and KD2 cell lines ( × 200). (**f**) BrdU+ cells in NT, KD1 and KD2 cell lines were quantified. *P<0.05. Data are presented as means±S.E.M.

To test whether DJ-1 can modulate LPC proliferation directly, LPCs were isolated from both WT and DJ-1 KO mice after feeding with DDC diet for 4 weeks. Those LPCs were subjected to *in vitro* culture followed by BrdU incorporation assay to examine the proliferation rate. Interestingly, although the LPCs of DJ-1 KO mice showed a proliferation disadvantage *in vivo*, there was no significant proliferation difference between WT and DJ-1 KO LPCs cultured *in vitro* (Figures 3a and b). To further confirm this observation, DJ-1 knockdown (KD) was performed in a liver epithelial progenitor cell (LEPC) line. Significant DJ-1 downregulation was confirmed in the KD group compared with the non-target control by both qPCR and western blotting (Figures 3c and d). Consistent with *in vitro* cultured LPCs, DJ-1 KD did not change the BrdU incorporation in LEPC lines (Figures 3e and f). These *in vitro* results strongly suggest that the impaired proliferation of LPCs in DJ-1 KO mice may be largely attributed to the LPC-associated niche changes caused by DJ-1 deficiency.

### The LPC-associated fibrogenic niche is impaired in DJ-1 KO mice

**Figure 4:**
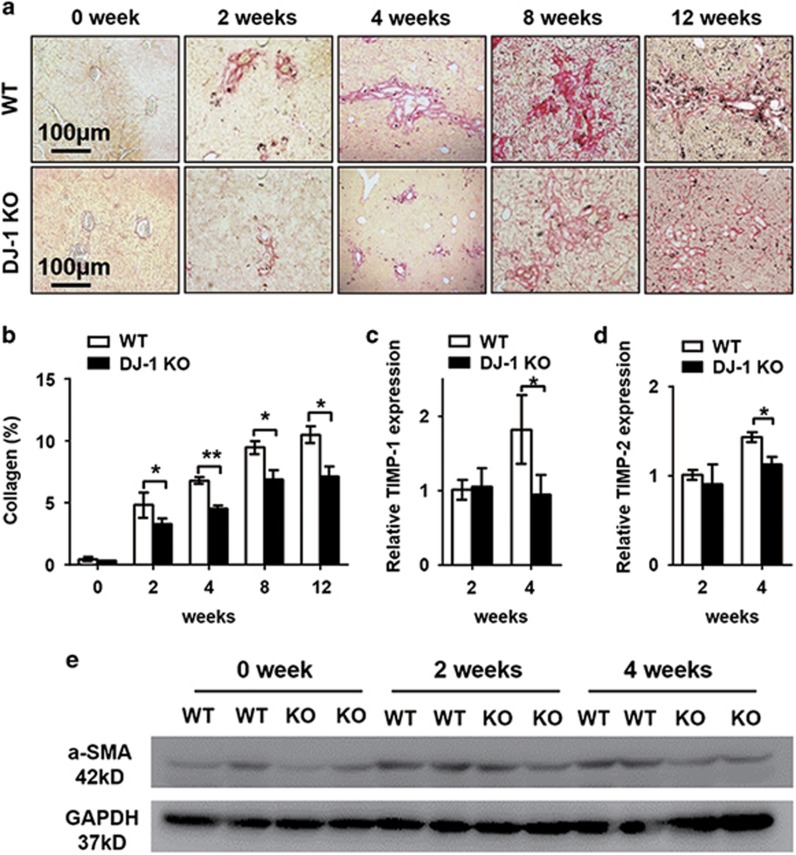
Liver fibrosis was impaired in DJ-1 KO mice. (**a**) Representative Sirus red staining of liver tissues from WT and DJ-1 KO mice after DDC feeding for different time periods. The original magnification is × 200. (**b**) The areas of Sirus red staining from panel (**a**) were quantified. (**c** and **d**) The relative TIMP-1 and TIMP-2 mRNA levels in liver tissues of WT and DJ-1 KO mice after 2 or 4 weeks of DDC diet feeding, respectively. (**e**) Western blotting analysis of *α*-SMA in liver tissues of WT and DJ-1 KO mice after DDC diet for 4 or 8 weeks. GAPDH (glyceraldehyde 3-phosphate dehydrogenase) expression was used as a loading control. *P<0.05 and **P<0.01. Data are presented as means±S.E.M.

A recent study indicated that extracellular matrix (ECM) remodeling occurs prior to LPC expansion and that *α*-SMA^+^ cells facilitate the parenchyma infiltration of LPCs in a CDE model,^26^ suggesting the requirement of a fibrosis niche for LPC expansion. Given that DJ-1 may affect LPC-associated niche to indirectly modulate LPC expansion, the fibrogenic microenvironment was examined in the DDC model. Although sirus red staining showed increased liver fibrosis in both WT and DJ-1 KO mice in a time-dependent manner, the intensity of liver fibrosis in DJ-1 KO mice was much weaker than that in WT mice (Figures 4a and b). Accordingly, the expression of *α*-SMA, a marker of activated hepatic stellate cell (HSC) and fibrogenic response, was also significantly lower in DJ-1 KO mice compared with WT mice (Figure 4e). In addition to the components of ECM, we also measured the expression levels of tissue inhibitor of metalloproteinase-1 (TIMP-1) and TIMP-2, two well-known profibrotic genes.^16, 27^ After 4 weeks of DDC exposure, the expression of TIMP-1and TIMP-2 was significantly lower in DJ-1 KO mice compared with WT mice (Figures 4c and d). Together, these results demonstrate that DJ-1 depletion jeopardized the formation of LPC-associated fibrotic niche in DDC model.

### The LPC-associated inflammatory niche is impaired in DJ-1 KO mice

**Figure 5:**
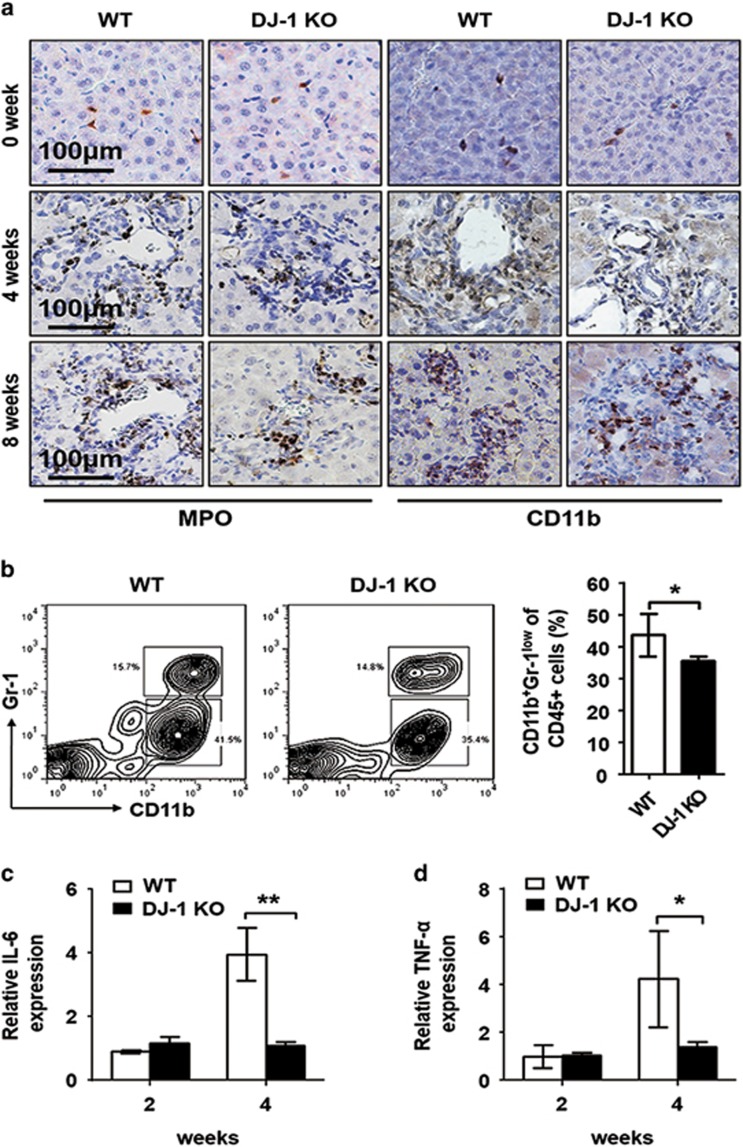
Macrophage infiltration into the liver was reduced in DJ-1 KO mice. (**a**) Representative IHC of anti-Myeloperoxidase (MPO) and anti-CD11b in liver tissues from WT and DJ-1 KO mice after DDC feeding for 0, 4 and 8 weeks. The original magnification is × 200. (**b**) Flow cytometric analysis of macrophage (CD11b^+^Gr1^low^) from 4 weeks of DDC diet feeding WT and DJ-1 KO mice (left). The percentage of macrophages in whole liver leukocytes (CD45^+^) (right). (**c** and **d**) The relative IL-6 and TNF-*α* mRNA level in liver tissues of WT and DJ-1 KO mice after 2 and 4 weeks of DDC diet feeding. *P<0.05 and **P<0.01. Data are presented as means±S.E.M.

To further explore the concept that the impaired LPC proliferation was dependent on the niche changes in DJ-1 KO mice, we next examined the liver inflammatory environment after DDC exposure. Consistent with the previous finding that liver-infiltrated macrophages can underpin the expansion of LPCs,^13^ we found significant less macrophage infiltration in the liver of DJ-1 KO mice compared with WT mice after 4 and 8 weeks of DDC feeding (Figure 5a). Meanwhile, the hepatic invading of neutrophils was comparable in both WT and KO mice (Figure 5a). We further confirmed this result by two-parameter flow cytometry to analyze the cell population in whole leukocytes isolated from the liver of WT and DJ-1 KO mice after 4 weeks of DDC feeding. As shown in Figure 5b, significantly greater hepatic infiltration of macrophages (CD11b^+^Gr-1^low^) was observed in WT mice compared with DJ-1 KO mice, whereas the infiltration of neutrophils (CD11b^+^Gr-1^high^) showed no great difference. As the pro-inflammatory cytokines IL-6 and TNF-*α* are well known to promote LPC activation, we also determined the hepatic levels of those two key cytokines. Although the levels of those two cytokines were comparable 2 weeks after DDC exposure, a significant induction of them was observed in WT mice but not in DJ-1 KO mice after 4 weeks of DDC feeding (Figures 5c and d). The expression of IL-6 and TNF-*α*, but not of Tweak, was also decreased in DJ-1 KO mice after 3 weeks of CDE diet feeding (Supplementary Figure S1b). These results indicate that DJ-1 deprivation deteriorated the formation of LPC-associated inflammatory niche in DDC model.

### HSCs from DJ-1 KO mice has the decreased ability to recruiting macrophages

**Figure 6:**
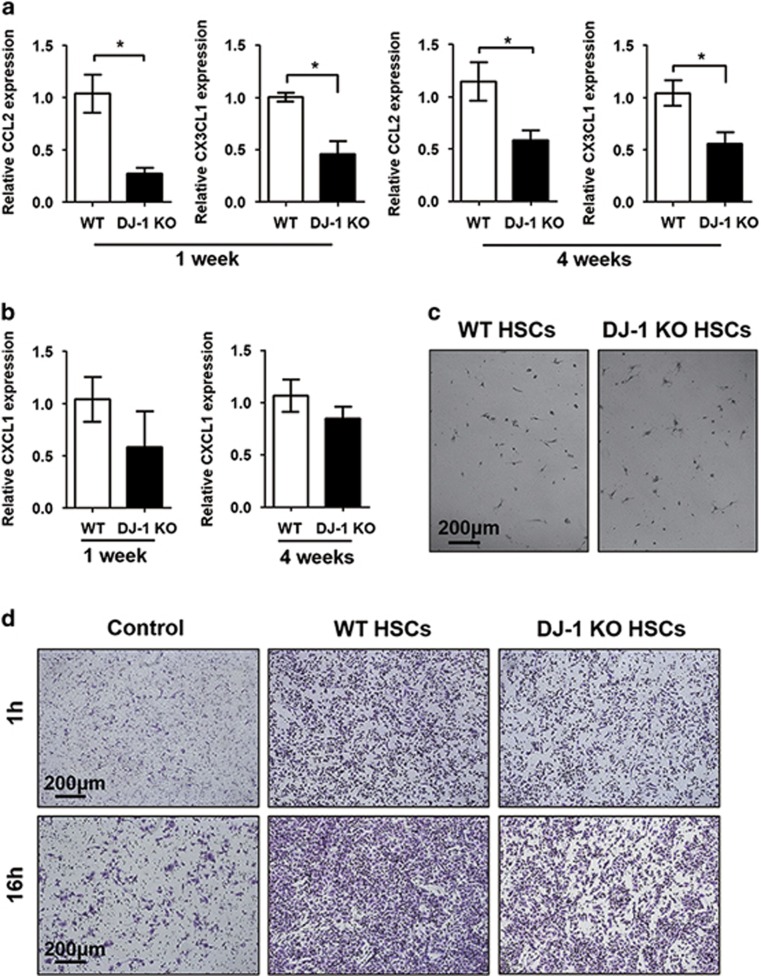
DJ-1 KO HSCs have impaired chemokine expression after DDC feeding. (**a**) The relative CCL2 and CX3CL1 and (**b**) CXCL1 expression in HSCs isolated from liver tissues after 1 and 4 weeks of DDC feeding. (**c**) The HSCs isolated from WT and DJ-1 KO mice with the same density was added into a Transwell chamber system. The original magnification is × 100. (**d**) Macrophage recruitment was detected after 1 and 16 h. *P<0.05. The original magnification is × 100

In addition to producing ECM, HSCs can also modulate the inflammatory environment through recruitment and activation of leukocytes by secreting several chemokines.^28, 29^ As we have shown that both LPC-associated fibrosis and inflammatory niches were impaired in DJ-1 KO mice after DDC feeding, we started to examine whether the reduced HSC activation contributes to the less macrophage infiltration in DJ-1 KO mice. We detected the chemokine levels that were reported responsible for macrophage (CCL2 and CX3CL1) and neutrophil (CXCL1) recruitment in HSCs after DDC feeding for 1 or 4 weeks. As shown in Figure 6a, the relative mRNA levels of CCL2 and CX3CL1 in HSCs were significantly lower in DJ-1 KO mice compared with their WT counterparts after 1 or 4 weeks of DDC diet. However, the expression of CXCL1 showed no great difference (Figure 6b). In order to further characterize whether the differential chemokine expression was responsible for the infiltration differences of macrophages in both WT and DJ-1 KO mice, we employed the Transwell chamber assay to determine the abilities of WT and DJ-1 KO HSCs in recruiting macrophages. Same amount of HSCs from WT and DJ-1 KO mice were used in this assay (Figure 6c). Compared with DJ-1 KO HSCs, significantly higher amount of macrophages were recruited by WT HSCs after either 1 or 16 h of co-incubation (Figure 6d). These results indicate that the less activation of HSCs may at least partially contribute to the impaired LPC-associated inflammatory niche in DJ-1 KO mice in a DDC model.

**Figure 7:**
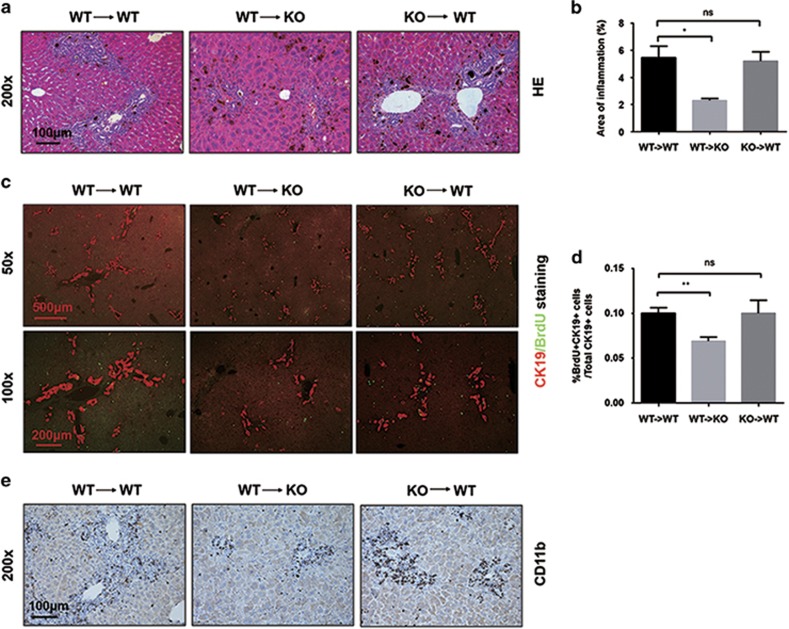
Immune cells do not contribute to the reduced LPC expansion in DJ-1 KO mice. (**a**) Representative hematoxylin–eosin staining of liver tissues of chimeric mice after 4 weeks of DDC exposure. (magnification × 200). (**b**) The areas of inflammation are quantified. (**c**) CK19 (red)/BrdU (green) double staining of liver tissues of chimeric mice fed with DDC diet for 4 weeks (× 50, upper panel; magnification × 100, lower panel). (**d**) Quantification of the percentage of BrdU/CK19 double-positive cells to CK19+ single-positive cells in the lower panel. (**e**) IHC staining for CD11b to detect infiltrating macrophages (magnification × 200). *P<0.05 and **P<0.01. NS, not significant

Given the possibility that DJ-1 may also affect the LPC-associated inflammatory niche by directly modulating the recruitment or activity of immune cells, we performed bone marrow transplantation experiments to examine whether immune cells are involved in the impaired LPC expansion in DJ-1 KO mice. To accelerate the Kupffer cell turnover, we depleted these resident macrophages by liposomal clodronate before lethal irradiation (Supplementary Figure S2a). Bone marrow was transferred from WT to WT, from WT to DJ-1 KO and from DJ-1 KO to WT mice in 4 h after irradiation (Supplementary Figure S2b). As shown in Figures 7a–e, the bone marrow transfer either from WT to DJ-1 KO or from DJ-1 KO to WT did not rescue or impair the DR, LPC expansion and hepatic macrophage infiltration in the recipient mice. These results strongly indicate that the liver-resident cells but not the immune cells mainly contribute to the impaired LPC-associated inflammatory niche in DJ-1 KO mice in the DDC model.

### CCL2 administration restores DR and hepatic macrophage infiltration in DJ-1 KO mice

To further elucidate whether CCl2 is the key mediator in DJ-1-related LPC expansion, DJ-1 KO mice were i.v. injected with recombinant CCL2 every other day 1 week after DDC exposure, when significant reduced CCL2 levels had been detected in HSCs derived from DJ-1 KO mice compared with WT mice. After 2 or 4 weeks of DDC feeding, mice were killed and the degree of DR was examined. As shown in Supplementary Figures S3a and b, administration of recombinant CCL2 significantly increased the DR and hepatic macrophage infiltration as demonstrated by HE and CD11b staining, respectively. These results indicate that reduced CCL2 secretion is attributable to decreased LPC response and hepatic macrophage infiltration in DJ-1 KO mice in the DDC model.

## Discussion

In the current study, we have demonstrated that DJ-1 expression is upregulated in the liver tissues of patients with chronic B viral or chronic C viral hepatitis, PBC, PSC, NAFLD, cirrhosis or HCC and has been associated with activated LPC proliferation. In addition, the expression of DJ-1 is also elevated in the LPC activation mice model caused by DDC diet feeding, whereas DJ-1 KO mice showed reduced LPC proliferation rate and impaired fibrosis scarring. Furthermore, results from our mechanistic studies suggest that DJ-1 promotes LPC response by indirectly co-modulating inflammatory response. We observed that LPCs-associated inflammation niche was decreased in DJ-1 KO mice, and the effects of DJ-1 on the LPC inflammatory niche was through modulating HSC chemokines production.

Currently, interaction between inflammatory niche and LPC proliferation have been well documented; many cytokines such as IL-6, TNF-*α*, Tweak, IL22 and IFN-*γ* have been shown to promote LPC proliferation.^6, 14, 15, 16, 18^ Our previous study have shown that DJ-1 directly regulate macrophage activity through modulating ROS production.^25^ In this respect, we hypothesized that DJ-1 may correlate with LPC proliferation via immune niche regulation. Indeed, we observed the decreased macrophages infiltration and reduced IL-6 and TNF-*α* expression in DJ-1-deficient mice, along with decreased collagen deposition, which is consistent with our unpublished data in a carbon tetrachloride-induced mice model. The relationship of inflammation and collagen deposition has also been well documented. Tirnitz-Parker *et al.*^16^ showed that these responses co-regulated the LPC proliferation in a murine model induced by CDE. Nevertheless, LPC proliferation correlates closely with the severity of fibrosis across a range of liver pathologies, including chronic HCV, alcoholic and nonalcoholic steatohepatitis, although there is still debate about cause and effect.

We found that DJ-1 KO mice showed reduced macrophages infiltration and lacked a DR to the DDC diet, along with a decreased liver injury. The decreased alanine transaminase and bilirubin (Supplementary Figures S4a and b) in DJ-1 KO mice may be caused by two reasons: the clearance rate in WT and DJ-1 KO mice may be different. On the other hand, the direct hepatocyte injury induced by DDC diet was different in mice of these two groups. Our previous study have shown that DJ-1 is a factor for NADPH oxidase-dependent ROS production,^25^ oxidase stress induced hepatocytes and ductal cells injury is the main reason for DDC-induced liver injury. We have stained ROS production in DDC-fed mice liver frozen sections and found lower ROS production in DJ-1 KO mice compared with WT mice (data not shown). In this model, we believe that DDC-induced lower ROS production in DJ-1 KO mice is the main reason for lower liver injury. However, because DJ-1 also have important role in macrophages function, whether the clearance rate is different or not in WT and DJ-1 KO mice need to be further studied. We also observed individual death in DJ-1 KO group mice but not in WT group mice after 4 weeks of DDC feeding, which is possible due to insufficient LPC activation to restore liver function in the early injury storm (data not shown). Generally, in the early stage (2–4 weeks), the LPCs proliferate very fast in both WT and DJ-1 KO mice. It is possible that the LPCs' quick evocation is required to restore the liver function in the condition of burst severe liver injury. Gradually, regenerative and differentiated hepatocytes or bile ducts maintain the normal function in WT mice, whereas impaired LPC expansion may cause impaired liver function restoration in DJ-1KO mice.

Previous study has reported the bipotential of LPCs to initiate primary HCC.^30, 31^ DJ-1, as a multifunctional protein, has been also reported to correlate with HCC.^32^ However, whether DJ-1 promotes liver progenitor cell transformation, which facilitate HCC tumorigenesis, remains largely unknown. In our study, in the DDC diet feeding model, the mice were fed for 1 year, and we did observe that a small proportion of mice developed tumor both in WT (1 in 5 mice) and DJ-1 KO mice (1 in 4 mice). It seems that DJ-1 deficiency could not prevent tumor incidence after DDC exposure for a long time period. However, the tumor size in DJ-1 KO mice is smaller than WT mice (data not shown). It is not surprising that LPCs participated in tumor-initiating process, which would be interesting to study the function of DJ-1 in HCC formation.

HSCs have been implicated to have a novel role in infiltrating immune cells through secreting chemokines during liver injury. To further study the mechanisms of DJ-1 on the DRs, we detected the chemokine levels that were reported responsible for macrophage (CCL2 and CX3CL1) recruitment in HSCs after DDC feeding^29^ and found that these chemokines were lower in DJ-1 KO mice. More importantly, the role of HSCs from WT and DJ-1 KO mice with DDC diet feeding in recruiting macrophages were analyzed using Transwell chamber assays *in vitro*. The HSCs from DJ-1 KO mice showed decreased ability to attract macrophages. By bone marrow transplantation experiments either from WT to DJ-1 KO or from DJ-1 KO to WT recipients, we find that immune cells do not change the *in vivo* phenotypes in both WT and DJ-1 KO mice in the DDC feeding model, suggesting that immune cells may not be attributable to the impaired DR in DJ-1 KO mice.

To further confirm that the reduced CCL2 levels contributes to the impaired DR in DJ-1 KO mice, recombinant CCL2 was introduced and restored DR and hepatic macrophage infiltration were observed in DJ-1 KO mice, suggesting that CCl2 is the key mediator in DJ-1-related LPC expansion.

To demonstrate whether DJ-1 also has a direct role in LPC expansion, an *in vitro* system was established. First, we isolated the primary LPC from WT and DJ-1 KO mice after 4 weeks of DDC feeding and measured the BrdU incorporation but did not find great difference between two groups. On the other hand, we also adopted siRNA targeting regime to intrinsically KD DJ-1 expression in a LEPC line. We observed that DJ-1 KD in LEPC lines does not show different proliferation rate. These results suggest that DJ-1 modulates LPC proliferation indirectly.

In the present study, DJ-1 has been shown to indirectly mediate LPC proliferation. DJ-1 appears to promote LPC proliferation through modulating LPC inflammatory niche and HSC chemokines production. Our results suggest that DJ-1 within the niche stimulates LPC proliferation. Inflammatory niche could promote LPC proliferation, and LPC proliferation could promote excessive matrix deposition, which further lead to fibrosis. These results support a hypothesis that DJ-1-targeted therapy might be useful in the future in the treatment of liver fibrosis.

## Materials and Methods

### Patient samples

Liver sections from patients with chronic disease were obtained from biopsy. The human sample study protocol was approved by the local ethics committee, and all of the patients provided signed informed consent.

### Animal experiments

C57BL/6 WT and DJ-1 KO mice were used as previously described.^25^ Proliferation of LPCs in WT and DJ-1 knockout (DJ-1 KO) mice was induced by feeding a 0.1% DDC (Sigma-Aldrich, Gillingham, UK)-containing diet or a CDE (Sigma) for a series of time periods. After 1 week of DDC diet feeding, DJ-1 KO mice received an intravenous injection of 1 *μ*g recombinant CCL2 every other day, meanwhile PBS injection was used as a vehicle control. BrdU (50 mg/kg) was administered by intraperitoneal injection 2 h before killing. All animal protocols were approved by the animal care and use committee in Stem Cell Research Center, Renji Hospital, School of Medicine, Shanghai Jiao Tong University, Shanghai, China.

### Cell line culture and small hairpin RNA KD

The murine-derived LEPC lines was kindly provided by Dr. Yongzhong Liu, which were cultured in DMEM containing 10% fetal bovine serum and antibiotics. The KD of DJ-1 vector was generated as previously described.^25^

### Gene expression analysis

Total RNA was extracted with the RNeasy Mini Kit (Qiagen, Hilden, Germany) according to the manufacturer's instructions and quantified by spectrophotometry (Nanodrop 2000, Thermo Scientific, Wilmington, DE, USA). Subsequently, 1 *μ*g total RNA was reverse-transcribed with the RevertAid First Strand cDNA Synthesis Kit (Thermo Fisher Scientific, Waltham, MA, USA). The relative expression levels of genes to *β*-actin were measured by real-time quantitative PCR with a ViiA 7 real-time PCR detection system (Applied Biosystems, Waltham, MA, USA).

### Histological analysis

Paraformaldehyde (4%)-fixed liver tissues were embedded in paraffin, and 5*-μ*m thickness sections were subjected to HE staining, Sirus red staining and IHC staining. Antibodies used in IHC staining included LPC-specific marker CK19 (Proteintech, Peprotech Group, Rocky Hill, NJ, USA), DJ-1 (Abcam, Cambridge, UK), Myeloperoxidase (Biocare Medical, Concord, CA, USA), CD11b (Abcam) and F4/80 (AbD Serotec, Kidlington, UK).

### Immunofluorescence

Heat-induced epitope retrieval was used in liver paraffin sections. For cultured cells, 70% ethanol fixation and 4 N HCl permeabilization were used before staining. Immunofluorescence double staining were stained with anti-CK19 (Proteintech) and anti-BrdU antibody (Cell Signaling Technology, Beverly, MA, USA). Slides were visualized by Alexa Flour 488-labeled anti-mouse antibody and Alexa Flour 564-labeled anti-rabbit antibody (Invitrogen, Carlsbad, CA, USA).

### Western blotting

Western blotting analysis was performed as previously described. Briefly, liver tissue and cultured cells were lysed by RIPA buffer (Thermo Scientific). A total of 40 *μ*g of protein was separated by SDS-PAGE and followed by transferred to nitrocellulose membrane (Amersham Pharmacia, Piscataway Township, NJ, USA). Protein bands were visualized by Immobilon western chemiluminescent HRP substrate (Millipore, Billerica, MA, USA) and detected with a Molecular Imager (Bio-Rad, Hercules, CA, USA). The primary antibodies against DJ-1 and *α*-SMA were purchased from Abcam and PCNA from Cell Signaling Technology. The horseradish peroxidase-conjugated secondary antibodies were obtained from Jackson Immuno Research Laboratories, Inc (Baltimore Pike, PA, USA).

### Primary culture of LPCs from DDC-induced mice

Livers from 2–4-week DDC-treated mice were used for primary liver progenitor cell isolation by a discontinuous 20–50% Percoll (GE Healthcare, Hatfield, UK) gradient centrifugation, as previously described.^6, 7^ Briefly, LPCs were isolated by a modified two-step perfusion protocol, accompanied by MACS-based anti-CD45 (Miltenyi Biotec, Cologne, Germany) negative selection.

### Flow cytometric analysis

Liver leukocytes were prepared after 4 weeks of exposure to DDC diet by a previously described protocol.^12^ Cell suspensions were incubated with monoclonal antibodies CD11b-PE (e-Bioscience, San Diego, CA, USA) and Gr-1-FITC (BD Bioscience, Franklin Lakes, NJ, USA) and then flow cytometric analysis was performed on a FACSCalibur instrument (BD).

### Primary HSC isolation from DDC-induced mice

Primary HSCs were isolated from WT or DJ-1 KO mice treated with DDC diet for either 1 week or 4 weeks similarly to a previously published procedure,^33^ with modifications. Briefly, liver was perfused *in situ* through the portal vein with 0.0375% type IV collagenase (Sigma) and 0.0004% DNAse I (Roche, Indianapolis, IN, USA) in GBSS buffer for 10–15 min. This was followed by postdigestion with 0.09% Pronase E (Roche), 0.1% collagenase and 0.0004% DNAse I at 37 °C for 20 min and a one-step centrifugation through a 12.5% density gradient of Percoll (Sigma) at 3000 r.p.m. for 18 min.

### Migration assay

The assay was performed as previously shown with minor modifications.^34^ Bone marrow-derived macrophages were isolated and stimulated with 40 ng/ml m-CSF for 7 days in 10% 1640 medium. Initially, 1 × 10^5^ macrophages were seeded into Transwell inserts with 8-*μ*m pores (Corning HTS Transwell-24, Corning Inc., Corning, NY, USA), which were placed into HSCs isolated from WT or DJ-1 KO mice treated with DDC diet for 1 week. The cells were incubated for either 1 or 16 h, after which the inserts were removed from the Transwells. Cells on the upper side of the insert were gently wiped with cotton buds, while the cells on the lower side of the insert were fixed in ethanol and stained with crystal violet.

### Bone marrow transplantation

We transferred the bone marrow from DJ-1 KO or WT mice (*n*=7–8/group) into 8-week-old recipients (all on the C57BL/6J background) after ablative *γ*-irradiation (9 Gy). Notably, we gave all recipients an injection of liposomal clodronate (100 *μ*l intravenously) before irradiation to deplete Kupffer cells and accelerate macrophage turnover, as described previously.^35^ After 4 weeks of reconstitution, mice were subjected to 4 weeks of treatment with DDC-supplemented diet feeding as described above. Peripheral blood cells from bone marrow chimeric mice were collected and identified by genotyping.

### Statistical analysis

All data were expressed as mean±S.E.M. To compare the values obtained from two groups, Student's *t*-test was performed. *P*-value <0.05 was considered significant.

## Abbreviations

BrdU: bromodeoxyuridine
CDE: choline-deficient ethionine-supplemented
CK19: cytokeratin19
DDC: 3,5-diethoxycarbonyl-1,4-dihydrocollidine
DR: ductular reaction
ECM: extracellular matrix
EpCam: epithelial cell adhesion molecule
HBV: hepatitis B virus
HCC: hepatocellular carcinoma
HSC: hepatic stellate cell
IFN-*γ*: interferon *γ*
IHC: immunohistochemistry
IL: interleukin
KD: knockdown
LEPC: liver epithelial progenitor cell
LPC: liver stem/progenitor cell
NAFLD: nonalcoholic fatty liver disease
ROS: reactive oxygen species
TIMP: tissue inhibitor of metalloproteinase
TNF-*α*: tumor necrosis factor-*α*
TWEAK: TNF-related weak inducer of apoptosis
WT: wild type
*α*-SMA: *α*-smooth muscle actin
