# Supplementary material for: DJ-1 deficiency attenuates expansion of liver progenitor cells through modulating the inflammatory and fibrogenic niches
Source: Cell Death Dis. 2016 Jun 9;7(6):e2257–. doi: 10.1038/cddis.2016.161 (PMC5143389; doi:10.1038/cddis.2016.161)
Supplement: Supplementary Figure Legends [file cddis2016161x1.doc]

**Figure S1. Decreased LPCs response in DJ-1 KO mice after CDE feeding.** (a) Immunofluoresence of LPC proliferation analysis (CK19, red) mixed with BrdU (green) in liver tissues of WT and DJ-1 KO mice (100x and 200x). (b) The relative expression of IL6, TNF-α and tweak in WT and DJ-1 KO mice. Data are presented as means ± S.E.M.

**Figure S2. Validation of bone marrow (BM) reconstitution.** WT or DJ-1 KO mice were injected with liposomal chlodronate (100µl or 200ul, i.v..) or vehicle. (a) immunohistochemistry staining for F4/80 to liposomal clodronate mediated Kuffer Cell depletion. (b) DJ-1 genotype was identified and seperated on 3% agarose gel by using genomic DNA from peripheral blood cells of recipient mice after BM transplantation from WT->WT(lane 1), WT->KO (lane2) KO->WT(lane 3),and by using genomic DNA from tails of WT(lane 4), DJ-1 KO(lane 5) as positive controls, and by using H2O as a negative control(lane 6).

**Figure S3. CCL2 administration restores DRs and macrophage infiltration in DJ-1 deficiency mice.** After DDC feeding for 1 week, mice were i.v. injected recombinant CCL2 every other day. Liver tissues were collected till overall DDC feeding period was reached for 2 or 4 weeks (a) HE staining to evaluate ductular reaction. (b) Immunohistochemistry staining for CD11b to assess invading macrophages (magnification 200x).

**Figure S4. WT and DJ-1 KO mice were fed DDC diet for various time periods**. (a) Serum ALT and (b) Bilirubin were measured. Data are presented as means ± S.E.M.
