# Supplementary figures and images for: DJ-1 deficiency attenuates expansion of liver progenitor cells through modulating the inflammatory and fibrogenic niches
Source: Cell Death Dis. 2016 Jun 9;7(6):e2257–. doi: 10.1038/cddis.2016.161 (PMC5143389; doi:10.1038/cddis.2016.161)

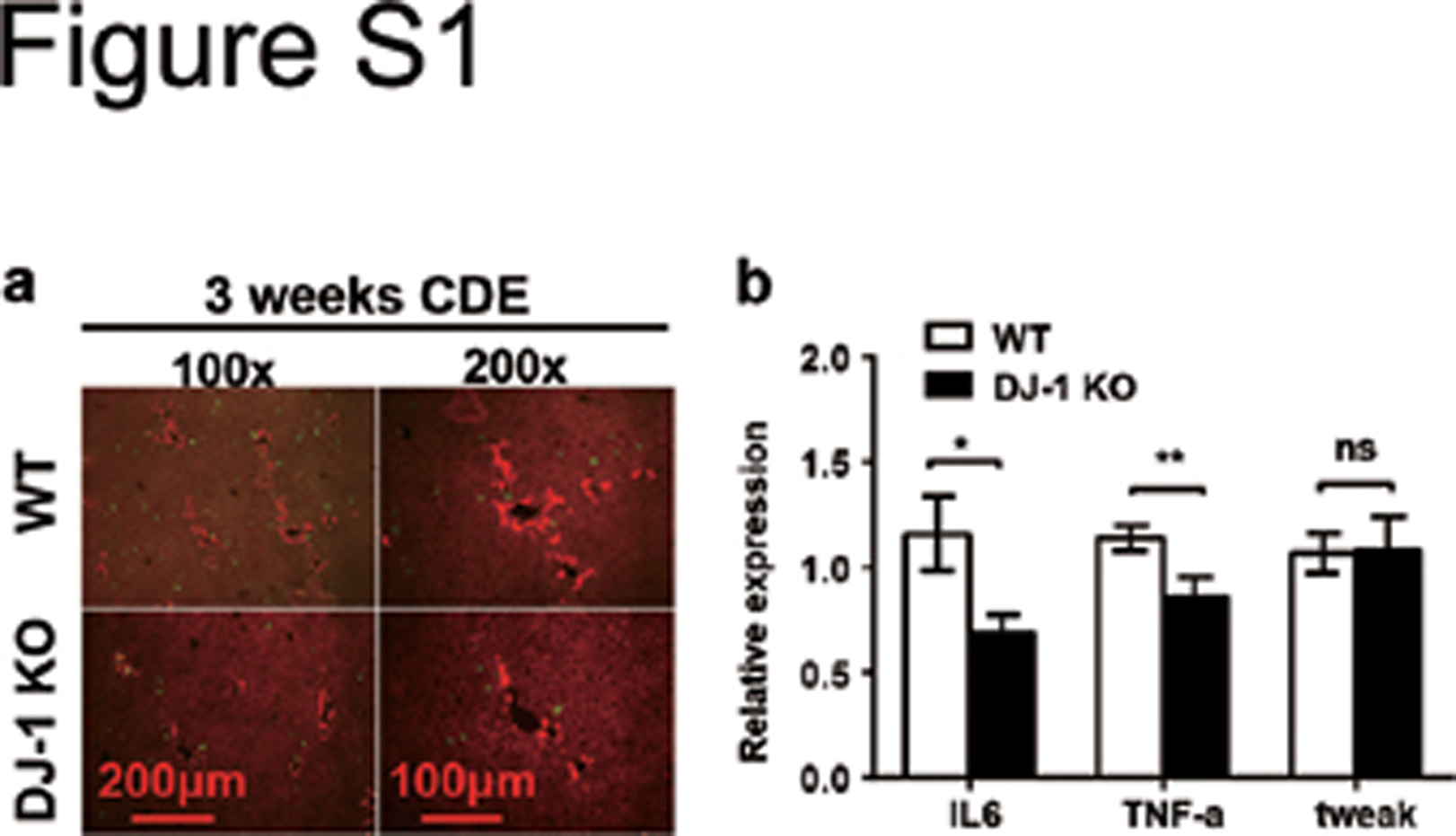

Supplement: Supplementary Figure 1 [file cddis2016161x2.tif]

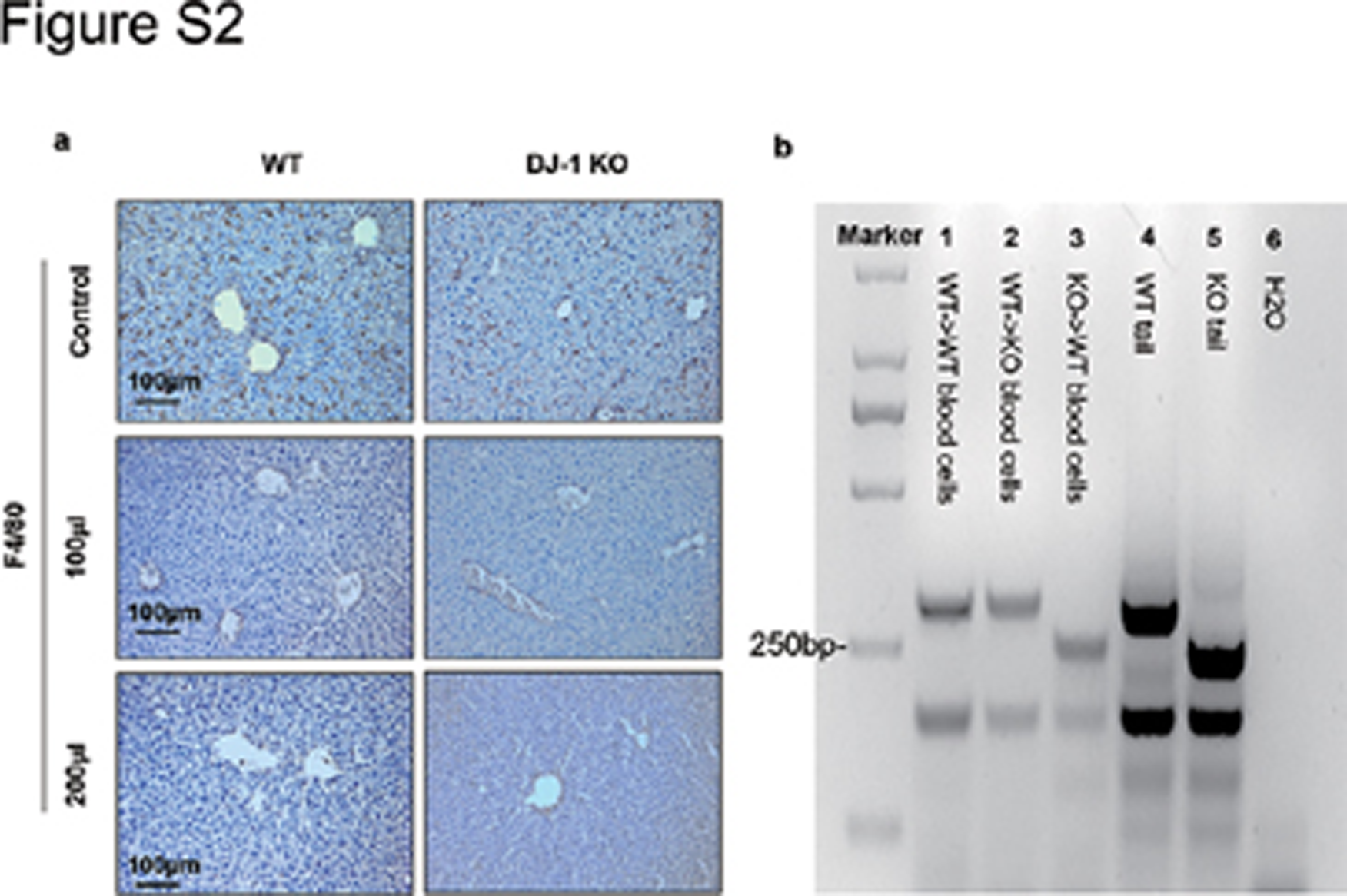

Supplement: Supplementary Figure 2 [file cddis2016161x3.tif]

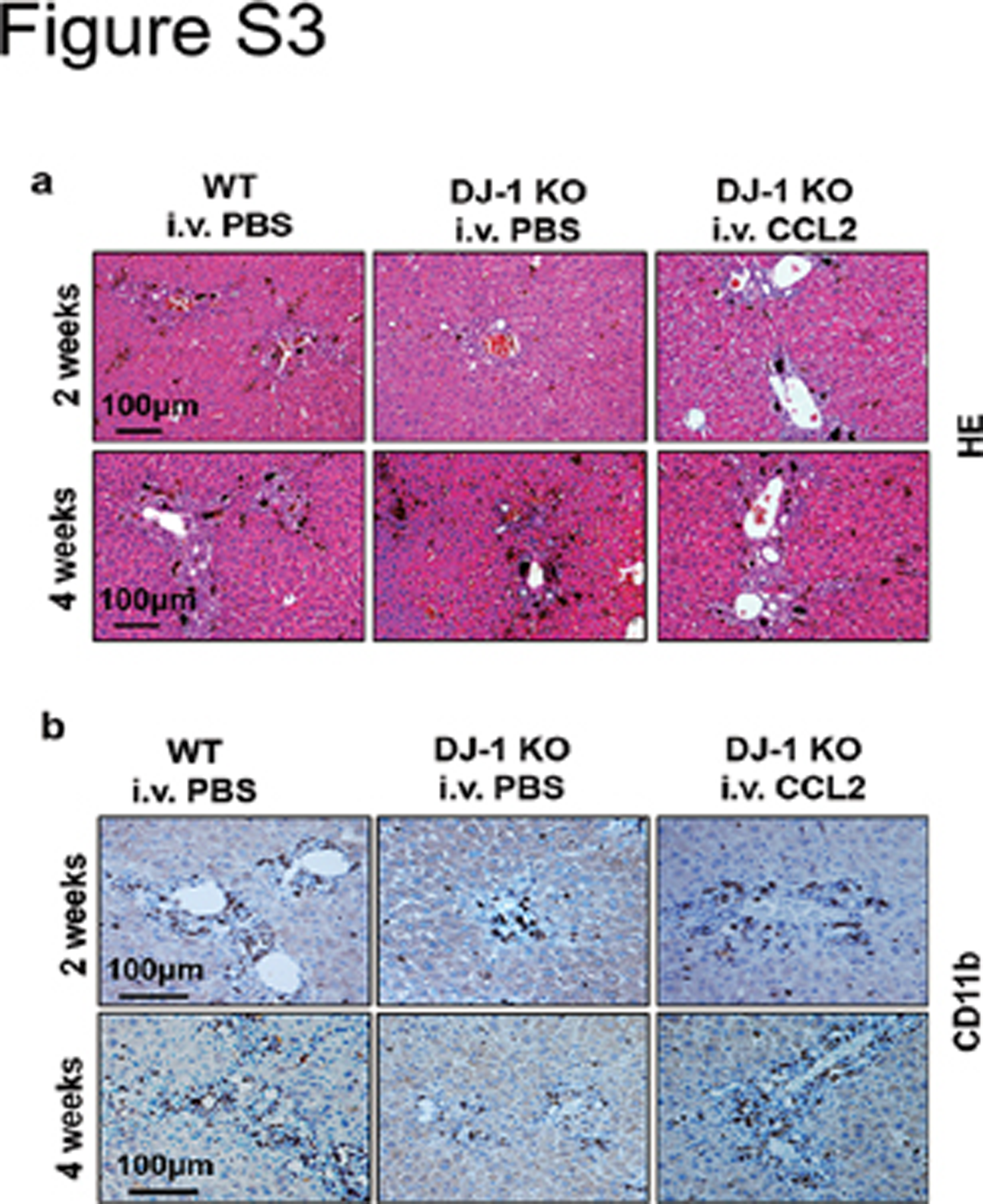

Supplement: Supplementary Figure 3 [file cddis2016161x4.tif]

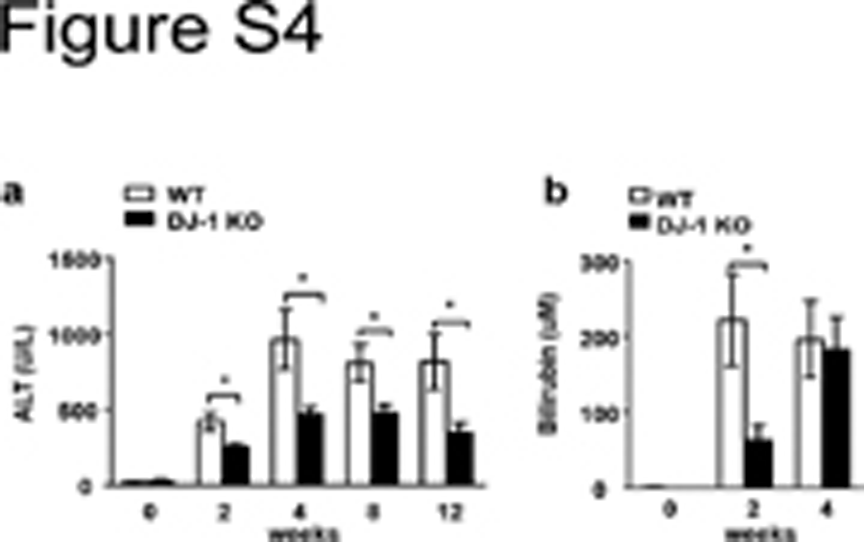

Supplement: Supplementary Figure 4 [file cddis2016161x5.tif]
